# Molecular imaging of HER2 receptor: Targeting HER2 for imaging and therapy in nuclear medicine

**DOI:** 10.3389/fmolb.2023.1144817

**Published:** 2023-03-02

**Authors:** Daniela Miladinova

**Affiliations:** Medical School, Saints Cyril and Methodis University, Skopje, North Macedonia

**Keywords:** molecular imaging, HER2 radiopharmaceuticals, trastuzumab, radionuclide therapy, theranostics

## Abstract

Targeting HER 2 for imaging and therapy in nuclear medicine has been used with a special emphasis on developing more powerful radiopharmaceuticals. Zirconium-89 plays an essential role in immune PET imaging so was used labeled with anti-HER2 antibody (Trastuzumab and Pertuzumab). Also there were attempts with other PET tracers as Cuprum-64 and Galium-68, as well as SPECT radiopharmaceuticals Indium-111 and Technetium- 99m. Regarding antibody pharmacokinetic that is not quite appropriate for imaging acquisition, several smaller molecules with shorter residence times have been developed. These molecules called nanobody, affibody, minibody do not compromize HER2 receptor affinity and specificity. Excess of Trastuzumab do not block the affinity of labeled affibodies. Silica nanoparticles have been conjugated to anti-HER2 antibodies to enable targeting of HER2 expressing cells with potential of drug delivery carry for antitumor agents and b(beta) or a(alfa) emitting radioisotopes commonly used for radionuclide therapy, as Iodine-131, Lutetium-177, Yttrium-90, Rhenium-188 and Thorium-277.

## Molecular imaging of HER2 receptor

The most frequently occurring cancer in women, breast cancer (BC), is responsible for almost 2 million new cases annually and approximately half a million related deaths worldwide. Female BC has surpassed lung cancer as the most common cancer worldwide (11.7%). One in eight women will develop BC at some stage during her lifetime (Deo et al., 2022).

The staging system most often used for BC is the American Joint Committee on Cancer TNM system, based on seven criteria: the tumor extent (T), spread to nearby lymph nodes (N), spread (metastases) to distant sites (M), grade (G), estrogen receptor status (ER), progesterone receptor status (PR), and HER2/neu status (HER2). Breast cancer is not a single disease (Rivenbank and O`Connor, 2013), and it appears to be very heterogeneous. It is characterized by different pathological features, disparate responses to treatments, and obvious differences in long-term patient survival.

Since its discovery in 1987 (Slamon et al., 1987), the HER-2/neu oncogene has been intensively investigated. Human epidermal growth factor 2 (HER2), also known as erbB-2 and neu, belongs to the ErbB or type I receptor tyrosine kinase family, together with human epidermal growth factors 1 (HER1), 3 (HER3), and 4 (HER4). The HER family includes transmembrane proteins, important for the activation of intracellular signaling pathways, as responses for extracellular signals. The receptor structure is very complex, made of an extracellular ligand-binding domain and transmembrane and intracellular tyrosine kinase domains. HER2 is overexpressed mainly in breast cancer but also in gastrointestinal, ovarian, and bladder cancer, and is associated with a highly aggressive infiltrating type of tumor prone to metastatic spreading (Iqbal and Iqbal, 2014).

Overexpression of HER2 accounts for 30% of invasive BCs and was associated with aggressive behavior and poor clinical outcomes before the introduction of anti-HER2 specific treatment (Slаmon et al., 2001). However, the development of monoclonal antibodies against the extracellular domain of HER2 significantly alters the natural history of HER2+ BC. Trastuzumab is a HER2 receptor blocker that has become the standard of care for the treatment of early-stage HER2+ BC and for metastatic BC (Arteaga et al., 2012; Mitri et al., 2012).

Immunohistochemistry (IHC) is important in the pathology of breast disease, as well as in other benign or malignant tumors, and has become a standard procedure for pre- and postoperative tissue specimens. The identification of estrogen, progesterone, Ki67, and HER2/neu has been recommended for clinical use in current pathology practice (Zaha, 2014). HER2 staining results are categorized as negative (0 and +1), equivocal (+2), and positive (+3). Currently, IHC is used as a screening test; tissues graded +2 on IHC undergo *in situ* hybridization (ISH), mainly fluorescence ISH (FISH) (Ahn et al., 2020). A combined test (HER2/CEP17) has been used as a more precise tool in recognizing HQE1R1. A combined test ratio (HER2/CEP17) is commonly used, as it is more precise in recognizing HER2 status (Wolf et al., 2018). Liquid biopsy has been used as an alternative to biopsy sampling and blood analysis for circulatory tumor cells (CTCs) and circulatory tumor DNA (ctDNA). CTCs have been analyzed using flow cytometry, nucleic acid extraction, and IHC, while ctDNA assessment has been done using different PCR-based methods. Liquid biopsies could be superior, as they represent the full burden of primary and secondary lesions, but have serious limitations such as insufficient amounts of cell-free DNA and contamination of tumor-cell-derived DNA with DNA from unrelated body processes.

HER2-targeted therapy, despite the great success of trastuzumab, remains a challenge. Patients are primarily resistant (intrinsic resistance), and some develop resistance during treatment (acquired resistance). HER2+ BC patients benefit from the addition of pertuzumab, which binds to domain II of the extrinsic part of HER2 receptor, unlike trastuzumab, which binds to domain IV. After the CLEOPATRA clinical trial, treating patients with progressive HER2+BC with a combination of trastuzumab, pertuzumab, and taxanes (docetaxel) has been recommended (Gu et al., 2016). A large group of patients expresses low levels of HER2 receptors. Widely used anti-HER2 therapies, which are assumed to be ineffective in patients with low-HER2 cancers, have not been used. Low expression is defined as a score of 1+ on IHC, or 2+ on IHC with negative result on ISH. Approximately 60% of HER2-negative metastatic BC patients express low levels of HER2. These low-HER2 tumors could be hormone-positive or -negative and vary in their reaction to systemic treatment. Hormone-positive and HER2-negative tumors have been treated with endocrine therapy and cyclin-dependent kinase 4 and -6 inhibitors. This treatment, apart from the side effects, leads to resistance in two years. Trastuzumab deruxtecan is an antibody drug conjugate consisting of a humanized anti-HER2 monoclonal antibody linked to topoisomerase I inhibitor and is currently being evaluated as a treatment in ongoing clinical studies. In recently published results from the DESTINY breast 04 trial, trastuzumab deruxtecan showed superior activity over chemotherapy in patients with low-expressed HER2 receptors. Thus, HER2 imaging is very important in patients without overexpression of HER2 receptors (Modi et al., 2022).

The treatment of BC has been based on tissue-based biomarkers including estrogen, progesterone, and HER2 receptor status. However, these markers cannot resolve intratumoral or intra/intermetastatic spatial heterogeneity. In addition, during specific therapy, cells change their dominant genotypes and treatment responses by developing resistance. Therefore, tumor heterogeneity is becoming a significant challenge, as it requires sequential biopsies. Unfortunately, repeated biopsies could overlook changes, as they are not taken at all metastatic sites at the same time (Shrijver et al., 2018). Based on the results of experimental mouse models, core needle biopsy (CNB) is associated with an increased incidence of pulmonary metastases. CNB creates an immunosuppressive microenvironment with higher levels of myeloid-derived suppressor cells, with reduced CD4 and CD8 T cells, as well as macrophages, suggesting immunosuppressive environments in the tumors. Additionally, higher expression of the epithelial mesenchymal transition genes SOX 4 and Ezh was observed, as well as increased numbers of CTCs (Mathenge et al., 2014). Molecular imaging could substitute and complement tissue-based biomarkers, as it allows noninvasive assessment of all body sites required by oncologists. It could be used for prognostic purposes (to distinguish tumors with good or poor prognoses), predictive purposes (to determine the most efficient available therapy), and pharmacodynamic purposes (to determine doses of novel therapies in clinical trials) (Ulaner et al., 2016a).

Targeting HER2 for imaging and therapy in nuclear medicine (NM) has been extensively used in recent clinical trials with a focus on developing new, easily available, and more powerful radiopharmaceuticals (RPs). Molecular imaging techniques substitute for invasive biopsy procedures, exhibiting an immense advantage over other available markers. They can easily distinguish patients who are responsive and non-responsive to HER2-targeted therapy from patients who are primarily resistant to anti-HER2 treatment. Imaging NM methods allow follow up of the patients, including monitoring the response and identifying the patients who are developing resistance to specific anti-HER2 therapy (Massicano et al., 2018). Imaging of HER2 expression has an advantage over FISH, as gene amplification does not always mean overexpression. Some somatic mutations are negative for gene amplification, while the tumor cells overexpress HER2 proteins and will be positive in molecular imaging with anti-HER2 RP (Bose et al., 2013).

## Radiolabeled HER2 for imaging

Many studies with different RPs (labeled intact monoclonal antibodies and other smaller molecules such as antibody fragments, affibodies, and nanobodies) have been published thus far. Radiolabeled trastuzumab and pertuzumab are promising tracers because of their high accumulation in HER2+ tumor tissue. However, they would be of limited effectiveness, as significant activity happens 3–5 days post-administration, too late for necessary treatment modification. Zirconium-89 (^89^Zr), a powerful tracer for immune PET imaging (Wright and Lapi, 2013), was used to label anti-HER2 antibodies (trastuzumab and pertuzumab). ^89^Zr exhibits appropriate physical characteristics, providing good spatial resolution, a low average energy of 396 keV, and a half-life of 3.27 days, which matches the long biological half-life of antibodies. Radiolabeling of intact antibodies with ^89^Zr has been done by modifying the native lysine side with desferrioxamine B (DFO). Using this chelator, Zr can be released from the radiometal to form Zr chloride and Zr oxalate; the latter significantly accumulates in bones. ^89^Zr trastuzumab had high uptake in the kidney, liver, and heart. However, clinical dosimetric studies (Laforest et al., 2016) have revealed the liver as a critical organ and very low, almost negligible, radiation doses in bone and bone marrow, showing the use of labeled monoclonal antibodies with ^89^Zr to be extremely safe. In addition, the resistance mechanisms that alter binding of trastuzumab to the HER2 receptor could be visualized by ^89^Zr trastuzumab PET scans (Laforest et al., 2016). ^89^Zr trastuzumab revealed high-quality images five days after injection, allowing for reproducible quantification of the uptake, suitable for follow-up. Although the antibodies could not cross the blood–brain barrier, brain metastases were visible, probably due to the disruption of the blood–brain barrier in the metastatic region (Dijkers et al., 2010). ^89^Zr trastuzumab was also used to detect HER2+ metastases in patients with HER2-negative primary tumors. Ulaner et al. revealed, in a group of nine scanned patients, five with HER2+ metastatic lesions detected by IHC and ISH, suggesting additional candidates for HER2 targeted therapy (2016b). Trastuzumab was the first FDA-approved monoclonal antibody for HER2+ BC treatment. However, producing the first affirmative results of treatment with trastuzumab became a challenge as a significant number of patients developed resistance. Another important limitation of trastuzumab is cardiotoxicity, as HER2 is expressed in the heart (Behr et al., 2001). For overcoming trastuzumab resistance, other antibodies such as pertuzumab, trastuzumab, emtasine (T-DM1), and the HER2 tyrosine kinase inhibitor lapatinib have been developed and approved for clinical use. Trastuzumab and pertuzumab were used in combination, showing good results in the treatment of HER2+ metastatic BC. In the ZEPHIR study, ^89^Zr trastuzumab was also used to predict the response to T-DM1using ^18^F-FDG and ^89^Zr trastuzumab PET/CT scans. Although using two PET/CT studies might be inconvenient for many centers, using imaging from early-phase drug trials to direct later trials successfully guides patients’ selections of appropriate therapies (Clark et al., 2016). Pertuzumab is a newer monoclonal antibody that binds to the HER2 receptor on different domains and has become more efficient than trastuzumab. Binding to different sites allows imaging during treatment with trastuzumab. In the first human study of ^89^Zr pertuzumab, Ulaner et al. compared dosimetry data with those obtained with ^89^Zr trastuzumab for six patients. Imaging should be of greatest importance for assessing HER2 heterogeneity and for targeting patients with metastases unsuitable for biopsy, as 20% of patients with HER2-negative primary tumors will develop HER2+ metastases (Ulaner et al., 2018). Positive results in the basic study have allowed wider use of ^89^Zr pertuzumab in larger group of patients, as demonstrated by ^89^Zr pertuzumab lesions in patients currently on trastuzumab therapy regimens (Ulaner et al., 2020). Tamura et al. (2013) reported imaging with ^64^Cu DOTA trastuzumab in six HER2+ BC patients. Using a ^64^Cu positron tracer with a shorter half-life of 12.7 h, optimal images could be obtained after two days. Additionally, radiation exposure with ^64^Cu DOTA trastuzumab was 2.5 times lower than with ^89^Zr trastuzumab. As this radiopharmaceutical has acceptable dosimetry and pharmacologic safety results, it might be used for the diagnosis of primary and secondary BC lesions and could predict the biologic effects of anti-HER2 antibodies, which would be helpful in choosing between treatment with anti-HER2 antibodies and HER2 thyrosine kinase inhibitors. In addition, ^64^Cu DOTA trastuzumab could be used in other HER2+ malignancies, such as advanced HER2+ gastric cancer (Tamura et al., 2013).

PET imaging is widely available in North America and Western Europe but not in other parts of the world. Therefore, the development of conventional tracers used for SPECT and SPECT/CT has undergone enormous research. ^111^In-labeled trastuzumab was used for SPECT/CT identification of HER2+ metastases. Perik et al. (2006) reported results for 17 patients scanned with ^111^In-DTPA trastuzumab in which all lesions detected previously were seen and other foci of increased uptake, in keeping with new lesions, were also confirmed. Apart from high sensitivity for lesions, the identification of cardiotoxicity could not predict trastuzumab-related cardiotoxicity, as heart uptake did not correlate with the subsequently developed left ventricular dysfunction (Perik et al., 2006).

Monoclonal antibodies are widely used for labeling purposes, as they are specific to their targets and their developed chemistry allows production in high yields for preclinical and clinical studies. However, their high molecular weights (150 kDa) and slow clearance rates make the best time for imaging 4–7 days after injection, which is too late for appropriate treatment decisions. Late images, obtained because of slow antibody clearance, are not quite appropriate for patient selection, and several smaller molecules with shorter residence times have been developed (Ge et al., 2021). These do not compromise HER2-receptor affinity or specificity. Excess trastuzumab does not block the affinity of labeled affibodies or tumor accumulation (Massicano et al., 2018). These molecules could be antibody fragments or variants such as Fab, F (ab’)2, single-chain Fv (scFv), diabodies, or minibodies with molecular weights from 25 to 100 kDa. Additionally, different nontraditional protein scaffolds such as domain antibodies, affibodies, nanobodies, and anticalins have been developed. Nanobodies are recombinant single (variable) domains of heavy chains with approximate weights of 12–15 kDa (Chakravarty et al., 2014). The main characteristics of these smaller peptides are their lower immunogenicity and shorter circulatory half-lives, different biodistributions and clearance pathways, and their ability to provide better-quality images. In addition, their chemical modifications are simpler, that is, more appropriate for routine clinical practice. Antibody-labeled fragments of trastuzumab were used to confirm mechanisms of resistance to trastuzumab in HER2+ BC, namely, increased expression of epidermal growth factor (EPGF). Razumienko et al. (2016) used the ^111^In-bispecific radioimmunoconjugate ^111^In DTPA Fab PEG_24_ for imaging tumor xenografts that express HER2, EGFR, or both receptors (Razumienko et al., 2016). Lam et al. (2017) reported production of a pertuzumab Fab segment labeled with ^64^Cu NOTA pertuzumab F (ab’)2 that can detect changes in HER2 expression one week after trastuzumab treatment in BT-474 xenografted mice (Lam et al., 2017). Generally, Fab fragments offer rapid blood clearance and better tumor contrast in the early phase but also diminish tumor uptake. Chains of Pro, Ala, and Ser (100–600 residues) were genetically fused to the C-terminus of the light chain by benchtop fermentation in *Escherichia coli*. Biodistribution was specific for protein tracers, with an intensive blood pool during the first 6 h after injection. The lesions, and some activity in the liver and the kidneys, were visible after 24 h. Hepatobiliary clearance was predominant among the activity in the gastrointestinal tract after 48 h. The tumor-to-blood ratio was 3.4, while the tumor-to-muscle ratio was 20, allowing good images to be obtained within an acceptable time period. Modification of the Fab fragments with PASylation leads to delayed blood clearance and adequately sensitive tumor accumulation (Richter et al., 2020).

Affibodies are medium-sized peptides (6.5 kDa) derived from nonimmunglobulin *α* helix-based scaffolds, with similar or even higher affinities than some molecular antibodies. Because of these characteristics, they are proven tracers for molecular imaging. Baum et al. (2010) reported the first human use of ^111^In ABY002 and ^68^Ga ABY 002 in patients with recurrent breast cancer. Labeled ABY revealed nine of 11 metastases seen on FDG PET scans. Rapid blood clearance of both radiopharmaceuticals allowed imaging 2–3 h after application. High uptake of radiolabeled ABY002 was seen in metastatic lesions, liver, and kidneys. Only two lesions in three patients could not be observed, one close to the kidney and one in the liver. High liver activity was unexpected, compared to experimental studies; one reason might be hepatocyte HER2 expression. Quantitative assessment using standard uptake values (SUVs) seems ideal for monitoring therapy during treatment with trastuzumab (Baum et al., 2010).

ABY 025 is a second-generation affibody with reduced nonspecific liver uptake that binds to domain III on the HER2 receptor, providing noncompetitive interaction with trastuzumab and pertuzumab and enabling rapid treatment follow-up. ABY 025 has been successfully labeled with ^68^Ga and ^111^In, so it could be used with both PET and SPECT (Sorensen et al., 2014).

The widespread interest in using ^99m^Tc for labeling means excellent nuclear decay characteristics, safety, and easy availability for routine use. Li et al. (2017) labeled a ^99m^Tc-HYNIC-H6F peptide that specifically accumulates in HER2+ tumors and might be promising for the diagnosis of HER2+ lesions. Apart from technetium availability, the main advantage of ^99m^Tc-HYNIC-H6F is its targeting of different regions of the HER2 receptor compared to trastuzumab. This provides a strong opportunity to monitor the therapeutic effects of trastuzumab, without competition at the binding area of the HER2 receptor (Li et al., 2017). Oroujeni et al. (2021) reported preclinical evaluation of second-generation affibody ^99m^TcZHER2:41071 with a 25–30 fold lower renal uptake and without compromising imaging properties or tumor uptake. Using the chelator GCCC on the ^99m^Tc-ZHER2 affibody, the authors developed a more appropriate second-generation affibody with the best tumor-to-blood ratio of all SPECT RPs already used (Oroujeni et al., 2021). Rainone et al. developed a ^99m^Tc radiolabaled nanosilica system, functionalized with a trastuzumab half chain that could be used as SPECT RP for the detection of HER2+ BC cells (Rainone et al., 2017). Nanoparticles could be also used for multimodality imaging. Yamaguchi et al. (2016) synthesized hyperbranched polyamidoamine (PAMAM), and generation three was grafted to the surface of amorphous silica nanoparticles. Afterward, processed nanoparticles (PCSNs) were conjugated to the fluorescence dye ICG, and finally ^99m^Tc was labeled to the PAMAM amino surface by double chelation with MAG3 (mercapto-acetyl-triglycin) and DTPA (dyethylen-triamine-pentaacetic acid) as chelating agents. Polyamidoamine-based functionalized silica nanoparticles (PCSNs) for multimodal imaging were synthesized with near-infrared fluorescence and were incorporated with indocyanine green (ICG) and technetium (^99m^Tc) to produce radioactivity (Yamaguchi et al., 2016).

## Radiolabeled HER2 for therapy

The theranostic concept is a pair of imaging and therapeutic agents that could be used to diagnose, treat, and monitor the treatment response. HER2 receptor is an important target for the theranostic principle because the same or similar radiopharmaceuticals could be used for diagnostic and therapeutic purposes, depending on the radionuclide used for labeling. High overexpression of HER2 in some tumors makes them good candidates for targeted radionuclide therapy, especially when the tumors are resistant to other HER2-directed therapies. Anti-HER2 probes have been successfully radiolabeled with various isotopes for targeted therapies (Gebhart et al., 2016). Although almost 90% of NM procedures are for diagnostic purposes, radionuclide therapy is permanently increasing. Radioimmunotherapy (RIT) for HER2+ BC has been developed, mainly with trastuzumab and therapeutic radionuclides, mainly the β-emitters ^131^ I, ^177^Lu, and ^188^Re; ^111^In (as Auger electron emitter); and the α-emitters ^225^Ac, ^227^ Th, and ^212^Pb. D’Huyvetter et al. (2021) reported the results of a phase I clinical trial with ^131^I GMIB-Anti-HER2-VHH1 in six healthy volunteers and three breast cancer patients. VHH1 is a single-domain antibody covalently linked to therapeutic ^131^I *via* the linker SGMIB (succinymidil-4-guanidino-methyl-3-iodobenzoate). Its favorable toxicity profile, with an absorbed dose to the kidney of 1.54 ± 0.25 mGy/MBq, and positive uptake in metastatic lesions, offers new therapeutic options for patients who have progressed on trastuzumab, pertuzumab, and trastuzumab emtansine (D’Huyvetter et al., 2021).

The development of biotechnology and the discovery of nanoparticles that can host different functionalities and be loaded with different therapeutic molecules makes possible the theranostic concept. Nanobodies could be used for cancer therapy as antagonistic drugs, targeting agents of effectors’ domains, and targeting moieties on the surfaces of the drug-delivery systems (Oliveira et al., 2013). The functional capability of the nanoparticles may provide useful theranostics for delivering antitumor agents. Silica nanoparticles have been conjugated to anti-HER2 antibodies to enable targeting of HER2-expressing cells with the potential of drug delivery for antitumor agents and *β-* or *α*-emitting radioisotopes commonly used for radionuclide therapy, such as ^131^I, ^177^Lu, ^90^Y, ^188^Re, and ^227^Th (Oliveira et al., 2013).

The combination of RIT and radiosensitizing chemotherapy has been shown to increase the effect of ^212^Pb trastuzumab. Targeted cytotoxicity of *α*-particle radiation with ^212^Pb trastuzumab has been used as an appropriate treatment for small tumors, disseminated disease, micrometastatic disease, and the eradication of malignant single cells. In addition to its cytotoxic effects, gemcitabine has been used as a radiosenzitizer. Pretreatment of tumor-bearing mice with two doses of gemcitabine before ^212^Pb trastuzumab significantly increased the survival of HER2-xenograft models (Milenic et al., 2007).

## Conclusion

The use of molecular imaging is still in the early stages of development. Before its approved acceptance as a clinically valuable molecular marker, imaging faces several challenges: proof of reproducibility in clinical studies and evidence of correlation between the test results and improved clinical outcome. Regarding the synthesis of the new molecules, further time is needed for their approval. Increasing numbers of new breast cancer patients, as well as the development of promising theranostic agents, will hasten the efforts of scientists worldwide in achieving the main goal of increasing the survival and improving the quality of life of BC patients.
